# Propylthiouracil-Induced Skin Vasculitis

**DOI:** 10.7759/cureus.27073

**Published:** 2022-07-20

**Authors:** Mariana S Almeida, Carina Ramalho, Francisco Gomes, Maria do Rosário Ginga, José Vilchez

**Affiliations:** 1 Internal Medicine Department, Centro Hospitalar Barreiro Montijo, Barreiro, PRT; 2 Pathology Department, Centro Hospitalar Barreiro Montijo, Barreiro, PRT

**Keywords:** adverse reactions, purpuric plaques, anca, antibodies, propylthiouracil, vasculitis

## Abstract

The use of propylthiouracil (PTU) is associated with the development of autoantibodies, namely, antineutrophil cytoplasmic antibodies (ANCAs), which are associated with the pathogenesis of ANCA-associated systemic vasculitis, most often related to the myeloperoxidase subtype (ANCA-MPO). The authors report the case of a 61-year-old woman on PTU for one year who was referred to Internal Medicine for a three-month evolution of painless non-blanching purple patches, non-pruriginous, involving the chest and legs. The autoimmunity revealed ANCA antibody positivity, with a cutaneous biopsy compatible with leukocytoclastic vasculitis/necrotizing vasculitis with involvement of small and medium-sized vessels. Clinical improvement was noted after the drug was discontinued, with the resolution of the analytical changes.

## Introduction

Propylthiouracil (PTU) is a drug of choice for the treatment of hyperthyroidism, a common autoimmune endocrine disease. One of its possible adverse effects is the development of antineutrophil cytoplasmic antibody (ANCA)-associated vasculitis (AAV), which may be limited to the skin or may involve internal organs such as the lungs and kidneys, resulting in systemic dysfunction. Clinical characteristics of PTU-induced AAV are similar to that of primary AAV (Wegener’s granulomatosis, microscopic polyangiitis, and Churg-Strauss syndrome) but usually have a milder course and better prognosis.

ANCAs are a useful tool to diagnose drug-induced vasculitis early in the disease course, but it is necessary for the clinician to be aware of the association between the potential pharmacological cause and the development of vasculitis. When a cutaneous biopsy is performed, it is usually compatible with leukocytoclastic vasculitis.

Here, we describe the case of a middle-aged woman with leukocytoclastic vasculitis with cutaneous involvement after a one-year course of PTU for the treatment of hyperthyroidism.

The authors consider it relevant to report this case because, although PTU-induced vasculitis is rare, the diagnosis can be challenging as the clinical picture may be indistinguishable from other forms of vasculitis, requiring a high degree of suspicion, particularly due to a possibly fatal outcome. It is important for clinicians to be aware of the potential adverse effects that can be easily treated with drug discontinuation, resulting in a probable favorable evolution. Appropriate immunosuppressive therapy should be administered only to patients with vital organ involvement to prevent progression to severe and irreversible disease.

## Case presentation

**Figure 1:**
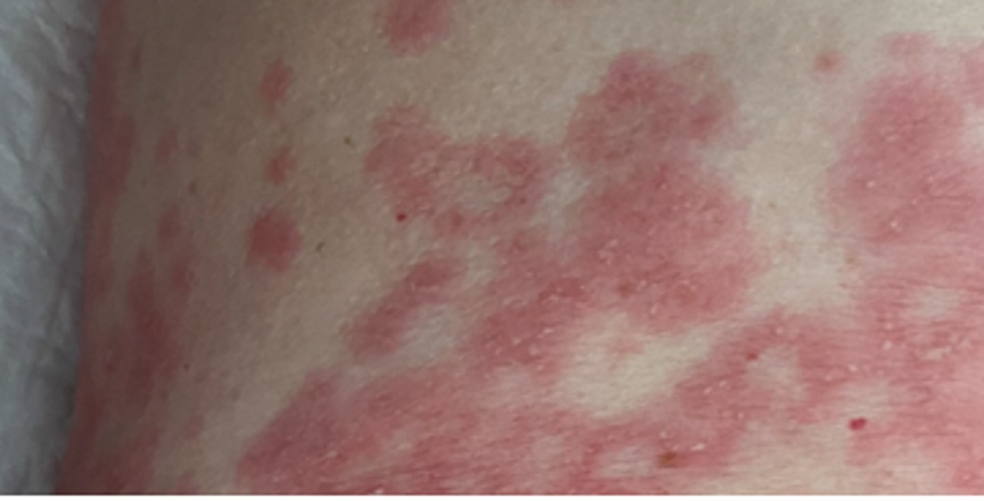
Erythematous, non-pruritic, purpuric plaques.

A 61-year-old woman presented with a history of epilepsy for 30 years, medicated since then with levetiracetam 500 mg twice a day, dyslipidemia controlled with food, and hyperthyroidism with one year of evolution, medicated since then with PTU 100 mg per day. She reported taking paracetamol occasionally for muscle pain in the last year but denied taking any other medication or herbal products. She was referred to the Internal Medicine Department due to a three-month evolution of erythematous, non-pruritic, purpuric plaques with approximate dimensions of 2-3 cm (Figure 1). The plaques began in the lower limbs and, after a week, in the chest, with pain and tenderness associated with palpation, as well as an appearance compatible with cutaneous vasculitis. She also reported knee arthralgias but denied fever, asthenia, weight loss, dyspnea, rhinitis, and cough. She had no family history of autoimmune diseases or asthma. On physical examination, she had non-pruritic, purpuric plaques, no signs of arthritis, no palpable masses, or organomegaly.

Blood tests showed normal blood cell counts, no changes in kidney function or urine test, increased sedimentation rate (35 mm), slightly increased C-reactive protein (16 mg/L), and perinuclear ANCA (p-ANCA) (134 RU/mL) and cytoplasmic ANCA (c-ANCA) (22.2 RU/mL) positivity. All other autoimmunity studies were negative, as well as viral serologies, namely, for human immunodeficiency virus, hepatitis B and C, cytomegalovirus, Epstein-Barr virus, and herpes simplex. Thyroid function tests were unremarkable (Table 1).

**Table 1:** Laboratory findings. ANA: antinuclear antibody; ANCA: antineutrophil cytoplasmic antibody; anti-dsDNA: double-stranded deoxyribonucleic acid antibody; anti-SSA/B: anti-Sjogren’s syndrome-related antigen A/B antibodies; CMV: cytomegalovirus; HIV: human immunodeficiency virus; free T4: free thyroxine

Laboratory tests	First consultation	First consultation after three weeks of drug suspension	Reference values
Hemoglobin (g/dL)	11.2	11.1	12–16
Leukocytes (/µL)	3,400	4,000	4,000–11,000
Eosinophils (/µL)	254	252	0–500
Lymphochites (/µL)	1005	1100	900–4,000
Platelets (/µL)	156,000	152,000	140,000–450,000
International normalized ratio	1.12	1.10	0.8–1.2
Creatinine (mg/dL)	0.6	0.7	0.6–1.2
Urea (mg/dL)	22	26	<50
C-reactive protein (mg/L)	16	7.7	<0.5
Sedimentation rate (mm)	35	12	<10
Thyroid-stimulating hormone (mU/L)	10.6	10.3	0.4–4.5
Free T4 (ng/dL)	0.54	0.89	0.5–1.8
Lupus anticoagulant, anti-cardiolipin and anti-beta2 glycoprotein antibodies	Normal		
Complement C3, C4	Normal		
Anti-dsDNA antibody	Negative		
Anti-SSA, anti-SSB	Negative		
ANA antibody	Negative		
p-ANCA (RU/mL)	Positive (134)	Positive (75)	<20
c-ANCA (RU/mL)	Positive (22.2)	Positive (23)	<20
Protein electrophoresis	No monoclonal pattern		
HIV, hepatitis B and C	Negative		
Antibodies for CMV, Epstein-Barr virus, and herpes simplex virus	Negative		

**Figure 2:**
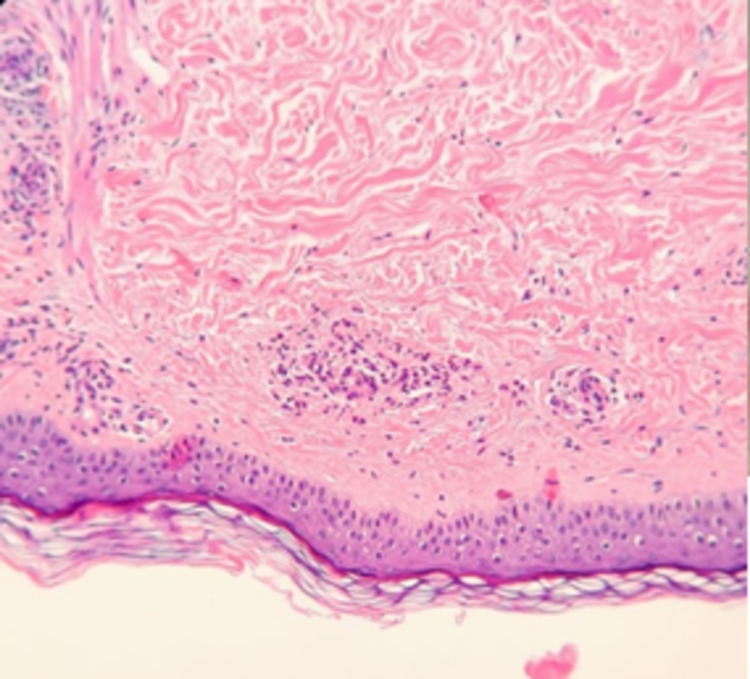
Skin biopsy compatible with leukocytoclastic vasculitis/necrotizing vasculitis, a pattern that is entirely compatible with the clinical context of positive ANCA vasculitis. ANCA: antineutrophil cytoplasmic antibody

**Figure 3:**
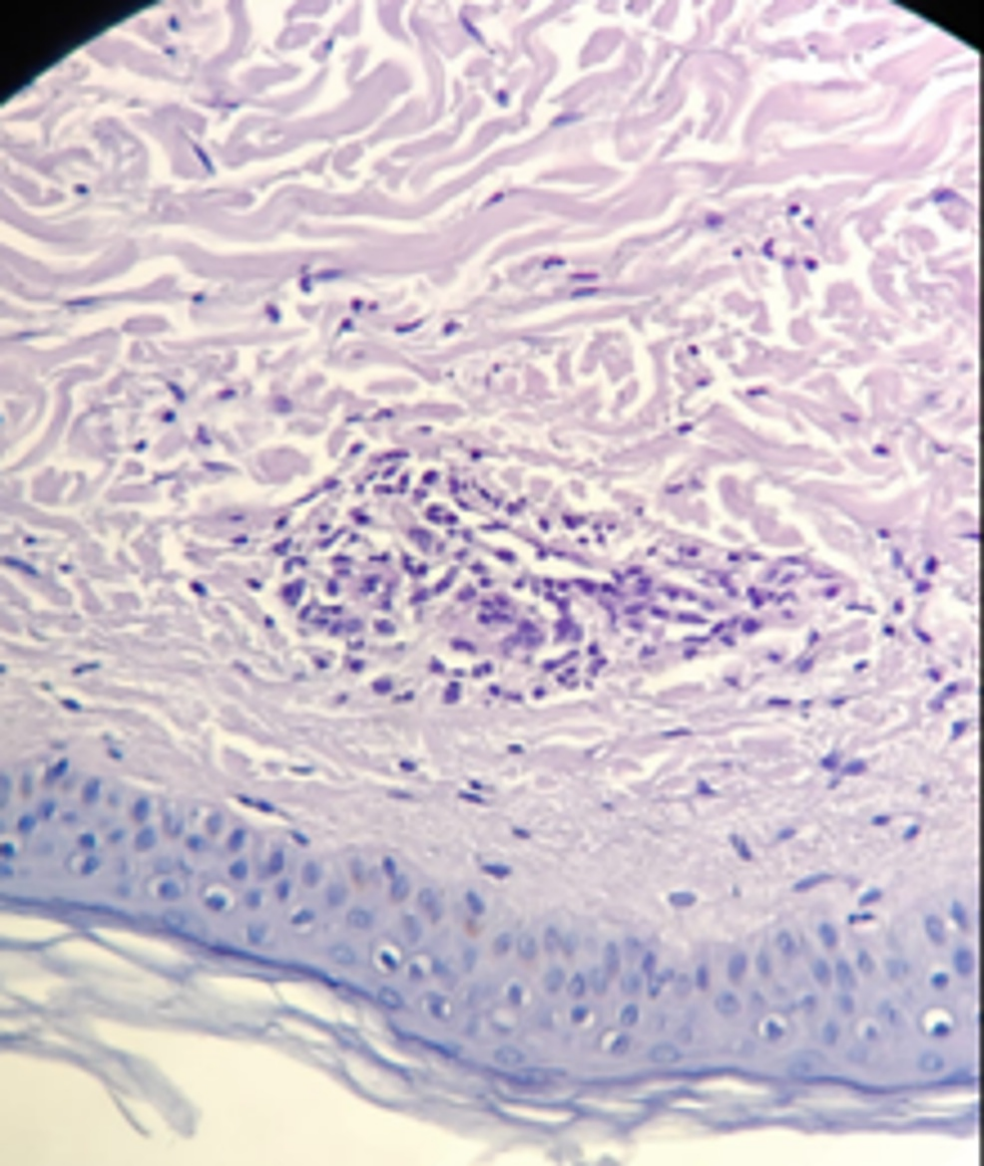
Skin biopsy compatible with leukocytoclastic vasculitis.

A chest teleradiography, nasolaryngoscopy, bronchoscopy, and thoracic-abdominal-pelvic computed tomography showed no alterations which allowed the exclusion of neoplasia and internal organ involvement in the context of primary or drug-induced vasculitis. For presenting skin lesions, a skin biopsy was done by the Dermatology team and showed leukocytoclastic vasculitis/necrotizing vasculitis with the involvement of small and medium-sized vessels in the superficial and deep dermis, a pattern fully compatible with the clinical context of positive ANCA vasculitis (Figures 2, 3).

After the above-mentioned diagnostic exams were performed, primary AAV, as well as neoplasia or infections, seemed unlikely as etiologies of the vasculitis. Given that the patient had PTU as her most recent drug initiation (one year) and after reviewing the literature on its possible association, the hypothesis of this being the cause of the vasculitis was raised, and it was decided to discontinue it. The patient was treated with an antihistamine (hydroxyzine, 25 mg per day), and the remaining medications she was taking were maintained. Because there was no involvement of vital organs, it was decided not to start corticoid therapy at this time.

During a re-evaluation visit after three weeks, the patient presented a clear improvement of the purpuric erythematous plaques, with no new lesions. Therefore, it was decided to just keep PTU suspended. Analytically, the p-ANCA value decreased (75.1 RU/mL) and the c-ANCA value remained unchanged (23 RU/mL). As the thyroid function values were normalized, she did not start any medication for hyperthyroidism, and her remaining medications were maintained. Due to the favorable clinical evolution, the diagnosis of ANCA-positive vasculitis induced by PTU was admitted. The patient was seen again at a follow-up visit at six months without any skin lesions and favorable analytical progress of autoimmunity tests. As a result of maintaining normal thyroid function, she did not restart any targeted therapy, and PTU remained suspended.

## Discussion

PTU is a thionamide widely used in the treatment of hyperthyroidism [1]. The most relevant side effects include agranulocytosis, hepatic toxicity, and vasculitis. Thionamide-induced vasculitis presents with arthritis, skin ulceration, vasculitic rash, acute renal failure, and lower and upper respiratory symptoms [2]. The first case of PTU-induced AAV was reported by Stankus et al. in 1992 in a patient with Graves’ disease who was treated with PTU. In 1993, Dolman et al. reported six patients with PTU-induced vasculitis [3]. PTU-induced vasculitis usually courses with the presence of ANCA, musculoskeletal manifestations, and cutaneous vasculitis [4]. A study by Zhao et al. found myeloperoxidase subtype (MPO)-ANCA positivity in 22.6% of patients treated with PTU, with evident vasculitis present in 28.6% [5]. ANCA is also an important serological marker of primary AAV such as Wegener’s granulomatosis, Churg-Strauss syndrome, and microscopic polyangiitis. PTU-induced AAV has similar clinical manifestations as primary AAV, and it is very difficult to differentiate between these two entities on the basis of clinical manifestations. Unlike primary AAV, which mainly occurs in elderly patients with a slight male predominance, patients with PTU-induced AAV are mainly young females. These differences are probably a reflection of the underlying disease because thyroid disease mainly involves young female patients. The disease severity of PTU-induced AAV is generally milder than that of primary AAV, provided that the drug is stopped sufficiently early. The overall prognosis of patients with PTU-induced vasculitis is also relatively better than the prognosis of patients with primary AAV. Vasculitis caused by PTU is usually ANCA-positive, more often with anti-MPO antibodies and the consequent p-ANCA pattern. It is known that the c-ANCA pattern is almost exclusively caused by anti-proteinase 3 (PR3) antibodies, in contrast to the p-ANCA pattern which may have anti-MPO, antilactoferrin, anti-human neutrophil elastase (HNE), or other antibodies as its origin [6]. In primary AAV, ANCAs usually recognize only one target antigen, namely, PR3 or MPO. In PTU-induced vasculitis, however, ANCAs usually recognize multiple target antigens, especially antigens other than PR3 and MPO [3]. Several cases have been described with anti-PR3 antibodies, resulting in ANCA with a cytoplasmic pattern in immunofluorescence (c-ANCA), as well as anti-lactoferrin and anti-HNE antibodies, whose detection is not routinely available [6]. These data may explain cases such as our patient because she had both positive ANCA, although with a greater positivity for p-ANCA, which corroborates the diagnosis of PTU-induced AAV.

Although the association between ANCA positivity and vasculitis is well known, its pathophysiology is not yet fully understood [7]. PTU has been shown to accumulate within neutrophils and bind to myeloperoxidase. The binding alters the configuration of myeloperoxidase and may promote the formation of autoantibodies in susceptible people [8]. Among patients with PTU-induced ANCA, only a fraction develop clinically evident vasculitis. Investigations into the differences between those who develop PTU-induced AAV and those who only have PTU-induced ANCA without clinically evident vasculitis have unraveled some of the risk factors for developing PTU-induced AAV, such as long-term exposure to PTU, serum ANCA against multitarget antigens, and serum positivity of MPO-ANCA, which were observed in our patient [3].

As already described, cutaneous manifestations of drug-induced vasculitis include palpable purpura and papules most commonly on gravity-dependent areas, such as the lower extremities and buttocks. Characteristically, PTU vasculitis occurs many months after the start of treatment, as was seen in our patient, and in some cases, it may manifest in just a few weeks. A similar case of PTU-induced AAV with purpuric plaques in the lower limbs following three years of PTU therapy was reported in a 46-year-old female by Milanez et al. [4]. Bilge et al. also reported a similar case in a 44-year-old male patient admitted with eruptions in lower extremities, with a biopsy compatible with leukocytoclastic vasculitis. The lesions disappeared after the withdrawal of PTU without any medication. He did not need any other antithyroid therapy as seen in our patient [9].

Because anticonvulsants can also cause leukocytoclastic vasculitis, a detailed temporal drug history was essential in this case. Rashid et al. performed a systematic review of cases of levetiracetam and cutaneous adverse reactions, which included leukocytoclastic vasculitis, with all reactions occurring with a duration of treatment between one week and one year, which allowed us to exclude the association between vasculitis and levetiracetam in this case [10].

The severity of PTU-induced vasculitis is variable and is limited to the skin; however, internal organs maybe involved, including the kidneys and lungs. Fatal cases have been reported even with limited cutaneous disease. Therefore, frequent monitoring for side effects is mandatory for patients on PTU therapy. Discontinuation of the drug should be the first step in the treatment and could lead to the complete resolution of symptoms. Typically, lesions resolve spontaneously within two to four weeks, but chronic or recurrent disease may occur in up to 10% of patients [1]. High doses of corticosteroid or cyclophosphamide may be needed in severe cases [2]. Although the optimal duration of immunosuppressive therapy is unknown, it is reasonable to gradually taper mediations and monitor clinical response [1]. Screening for ANCA is advisable in patients receiving long-term (more than 18 months) antithyroid medication.

## Conclusions

PTU is a drug used to treat hyperthyroidism. One of the possible adverse effects is the development of vasculitis, with the presence of ANCA-positive antibodies. The association between ANCA and vasculitis is well known, but its pathophysiology is not yet fully understood.

Associated vasculitis affects the skin and internal organs such as the lungs and kidneys. The case report highlights PTU-associated vasculitis as a possible side effect, requiring strong clinical suspicion. Prompt discontinuation of the drug is usually sufficient; however, severe cases might need oral corticosteroids.
